# The Impact of Genetic Susceptibility to Systemic Lupus Erythematosus on Placental Malaria in Mice

**DOI:** 10.1371/journal.pone.0062820

**Published:** 2013-05-10

**Authors:** Michael Waisberg, Christina K. Lin, Chiung-Yu Huang, Mirna Pena, Marlene Orandle, Silvia Bolland, Susan K. Pierce

**Affiliations:** 1 Laboratory of Immunogenetics, National Institute of Allergy and Infectious Diseases, National Institutes of Health, Rockville, Maryland, United States of America; 2 Biostatistics Research Branch, National Institute of Allergy and Infectious Diseases, National Institutes of Health, Rockville, Maryland, United States of America; 3 Comparative Medicine Branch, National Institute of Allergy and Infectious Diseases, National Institutes of Health, Rockville, Maryland, United States of America; Institut Jacques Monod - UMR 7592 CNRS - Université Paris Diderot, France

## Abstract

Severe malaria, including cerebral malaria (CM) and placental malaria (PM), have been recognized to have many of the features of uncontrolled inflammation. We recently showed that in mice genetic susceptibility to the lethal inflammatory autoimmune disease, systemic lupus erythematosus (SLE), conferred resistance to CM. Protection appeared to be mediated by immune mechanisms that allowed SLE-prone mice, prior to the onset of overt SLE symptoms, to better control their inflammatory response to *Plasmodium* infection. Here we extend these findings to ask does SLE susceptibility have 1) a cost to reproductive fitness and/or 2) an effect on PM in mice? The rates of conception for WT and SLE susceptible (SLE^s^) mice were similar as were the number and viability of fetuses in pregnant WT and SLE^s^ mice indicating that SLE susceptibility does not have a reproductive cost. We found that *Plasmodium chabaudi* AS (*Pc)* infection disrupted early stages of pregnancy before the placenta was completely formed resulting in massive decidual necrosis 8 days after conception. *Pc*-infected pregnant SLE^s^ mice had significantly more fetuses (∼1.8 fold) but SLE did not significantly affect fetal viability in infected animals. This was despite the fact that *Pc*-infected pregnant SLE^s^ mice had more severe symptoms of malaria as compared to *Pc*-infected pregnant WT mice. Thus, although SLE susceptibility was not protective in PM in mice it also did not have a negative impact on reproductive fitness.

## Introduction

It is estimated that in Africa more than 125 million pregnant women are at risk for *Plasmodium falciparum* infections leading to placental malaria (PM). PM has important consequences for both mother and fetus, resulting in low birth weight and 100,000 infant deaths annually [1]. Although the exact mechanisms underlying the pathology of placental malaria are incompletely understood, parasite sequestration in the placenta leading to impaired trophoblast invasion, placental vascular dysfunction and inflammation play roles in the disease [2], [3]. Similarly, another form of severe malaria, cerebral malaria (CM), an important cause of mortality in African children, is caused by a combination of ischemia associated with sequestration of parasites in the brain and microvascular damage, edema, blood brain barrier breakdown and immune involvement including inflammation [2], [3].Thus, both CM and PM have many features of uncontrolled inflammation leading to tissue damage and subsequent pathology.

Because CM and PM kill young children and pregnant women, it is likely that genetic mutations that result in increased survival in CM and PM, including mutations resulting in better control of inflammation, have been fixed in the African genome. Paradoxically, for the systemic autoimmune disease, systemic lupus erythematosus (SLE), a disease of uncontrolled inflammation, African American women are 6–8 times more at risk as compared to American women of European descent, suggesting that the African genome carries SLE-susceptibility genes [4]. Because the prevalence of SLE in Africa is extremely low [5]–[7], it has been hypothesized that SLE-susceptibility genes are beneficial in controlling severe malaria but in the absence of malaria act to promote inflammation [6], [8]. These observations led us to test the hypothesis in mice that genetic susceptibility to SLE was protective in severe CM. We infected mice that were susceptible to SLE (SLE^s^ mice) due to a deficiency in the immune inhibitory receptor, FcγRIIB [9], with *P.* berghei ANKA (*Pb* ANKA), a parasite that causes lethal CM [10]. SLE^s^ mice develop lethal SLE by 9 months of age, however, when infected at 4 to 6 weeks of age before the onset of any overt autoimmune inflammation, the SLE^s^ mice were protected from CM [11]. Protection appeared to be mediated by mechanisms that allowed the SLE^s^ mice to better control inflammation possibly through the heightened production of the anti-inflammatory cytokine IL-10. Consistent with this conclusion, *Pb* ANKA-infected SLE^s^ and WT mice were equally susceptible to death caused by severe anemia that did not involve inflammation. Relevant to these observations in mice, a human FcγRIIB allele that encodes a polymorphism in the transmembrane domain of the receptor that results in loss of function and is associated with SLE (OR = 1.73), is significantly more common in Africans and is associated with protection from severe malaria in African children [12].

Here we extend our earlier findings to determine the impact of SLE-susceptibility on PM in a mouse model to determine if SLE-susceptibility has an impact on reproductive fitness and if SLE-susceptibility is protective in PM. We evaluated SLE^s^ mice infected with non-lethal parasite *Plasmodium chabaudi chabaudi* AS (*Pc*) that induces a disease which is similar to PM, involving sequestration of infected red blood cells in the placenta and local inflammation that leads to fetal loss [13].

## Materials and Methods

### Ethics Statement

All experiments were approved by the National Institute of Allergy and Infectious Diseases Animal Care and Use Committee.

### Animals

Female 11 weeks old C57BL/6 were obtained from Jackson Laboratories and allowed to acclimate in the animal facility until the experiment was performed. SLE^s^ mice, B6.FcγRIIB[KO] (TAC264), were bred at the NIAID animal facility. Based on the literature, these mice do not develop active SLE disease before 6 months of age [9].

### Malaria Infections and Treatments

A total of 36 female SLE^s^ mice and 36 female C57BL/6 B6 mice were used in the experiment. To investigate the effect of SLE susceptiblity on pregnancy and placental malaria, 20 mice from each group were inoculated with *P. chabaudi chabaudi* AS (*Pc*) infected red blood cells (iRBCs). Mice were mated in harems containing one male and two females. Each day, female mice were checked for the presence of vaginal plugs to determine copula. The day that animals were determined to be plugged was designated day 0 of pregnancy.

Parasites were obtained from donor C57BL/6 mice that were infected with thawed parasite stocks. The infected blood used for inoculums was diluted to the appropriate concentration using phosphate-buffered saline. On Day 0, 20 animals were either infected with 1×10^4^ *Pc* iRBCs i.v. or sham injected with 100 µL phosphate-buffered saline i.v. The inoculum dose and route was chosen in order to obtain peak parasitemia (estimated to be at day 10) close to the time when placentas are formed. In all cases after identification of a vaginal plug, females were separated from males and manipulation avoided for 6 days in order to prevent stress-induced blastocyst implantation failure. Body weight, parasitemia, and hemoglobin data were collected daily between day 6 and 8 after mating. Parasitemia in infected mice was quantified by examining Giemsa-stained thin blood smears. Hemoglobin concentration was measured with a HemoCue Hb 201+ using blood from the tail tip. Hematology parameters were determined before animals mated and on day 8 post-conception using a Hemavet 950 FS system (Drew Scientific). All mice were evaluated daily for the presence of bloody vaginal discharge. On day 8 after mating, animals were sacrificed by CO_2_ asphyxiation, their uteri were dissected and the number of fetuses counted.

### Pathology

Uterine tissue from all mice used in this study (n = 72) was fixed in 10% neutral buffered formalin for 48 h and then transferred to 70% ethanol where the tissue was stored until further processing. After fixation specimens were embedded in paraffin and sections, cut at 5 µm, were stained with H&E. All sections were reviewed by a veterinary pathologist blinded to the design of the experiment.

### Data Analysis

A total of 40 animals (20 per group) were inoculated with parasites. 20/20 (100%) SLE animals that were inoculated got infected, while 15/20 (75%) controls got infected. Except where noted, the analyses reported in this paper were performed after excluding inoculated but uninfected mice (n = 5). Independent continuous outcomes were summarized using the means and standard deviations, and were analyzed using Student’s t-test and the linear regression. Binary data were summarized using percentages, and were analyzed using Fisher’s exact test and logistic regression. The generalized estimating equation (GEE) method with an exchangeable correlation structure as the working assumption was applied to analyze longitudinal continuous variables (i.e. parasitemia, hemoglobin concentration, body weight, and hematological parameters) in order to account for correlation among multiple measurements for the same mouse. Count data, such as total fetuses and viable fetuses, were analyzed using the Poisson regression with robust standard errors, thus allowing for the violation of the assumption that the variance equals the mean for the Poisson distribution. All *P* values were two-sided and P values of less than 0.05 were considered to be statistically significant. Data were analyzed using STATA (version 10.0; STATA Corporation, College Station, Texas, US).

## Results

### Effects of SLE Susceptibility on the Severity of *Pc* Infections

**Figure 1:**
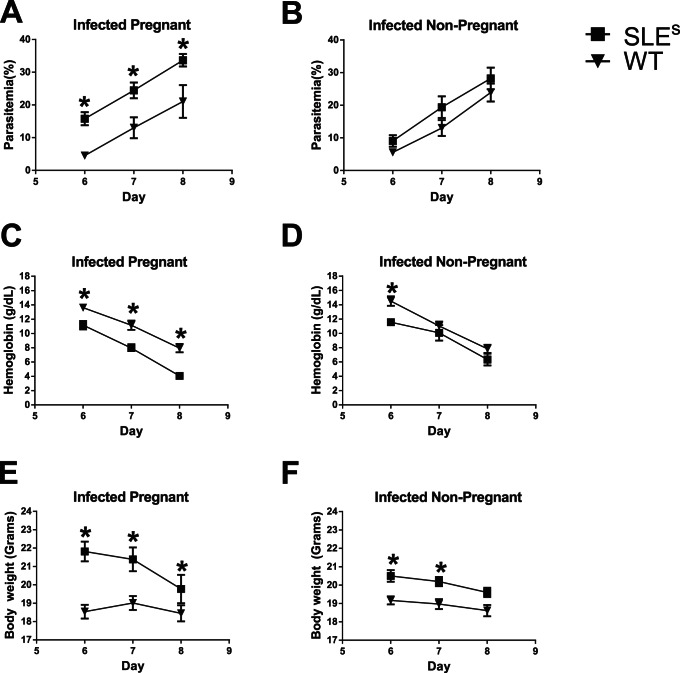
Effects of SLE susceptibility and malaria on parasitemia, hemoglobin and body weight. (A–B) Peripheral parasitemia were determined at days 6, 7 and 8 post-conception. (C–D) Blood hemoglobin concentrations were determined at day 6, 7 and 8 post-inoculation. Data are stratified according to pregnancy and infection status. (E–F) Body weight was determined at days 6, 7 and 8 post-conception. Data was stratified according to pregnancy and infection status. n = 15 (WT) and n = 18 (SLE^s^) for parasitemia; n = 30 (WT) and 34 (SLE) for hemoglobin and body weight.

To evaluate the effects of SLE susceptibility on the severity of *Pc* infections, we measured parasitemia, hemoglobin, and body weight in infected and pregnant and nonpregnant WT 6, 7 and 8 days after *Pc* infection and in control uninfected mice. n = 15 (WT) and n = 18 (SLE^s^) for parasitemia; n = 30 (WT) and 34 (SLE) for body weight and hemoglobin. A generalized estimating equation (GEE) analysis with indicator variables for SLE-susceptibility, pregnancy, time after infection, and the corresponding two-way and three-way interactions as covariates were performed for each parameter (Table S1). As expected, parasitemia increased with time in all infected mice (Figure 1A, B). The GEE analysis showed that pregnant infected SLE^s^ mice had significantly higher parasitemias level as compared to pregnant infected WT mice at day 6 (11.1% [95% CI, 3.6–18.7] higher; P = 0.004), day 7 (10.0% [95% CI, 2.4–17.6] higher; P = 0.01) and day 8 (7.8% [95% CI, 0.2–15.4] higher; P = 0.04) (Figure 1A). In contrast, non-pregnant, infected SLE^s^ and WT mice had parasitemias that were not significantly different (Figure 1B) indicating that the observed increase in parasitemias in SLE^s^ versus WT mice were pregnancy related.

Hemoglobin levels, a surrogate of red blood cell numbers, are predicted to drop as parasitemias rise during *Pc* infection. Applying the GEE analysis to uninfected mice (n = 16 for SLE^s^ mice and n = 16 for WT mice), adjusting for the time variables and pregnancy status, showed that SLE-susceptibility alone did not have a significant effect on hemoglobin levels. The GEE analysis showed that pregnant, *Pc*-infected SLE^s^ mice had significantly lower hemoglobin levels as compared to pregnant infected WT mice at day 6 (2.6 g/dL [95% CI, 0.6–4.5] lower; P = 0.01), day 7 (2.9 g/dL [95% CI, 1.0–4.8] lower; P = 0.003) and day 8 (3.5 g/dL [95% CI, 1.6–5.4] lower; P<0.001) post *Pc* infection (Figure 1C). The decrease in hemoglobin levels in infected SLE^s^ versus WT mice appeared pregnancy related as non-pregnant *Pc*-infected SLE^s^ and WT mice had hemoglobin levels that were not significantly different at day 7 and 8 post *Pc* infection.

Total body weight also tends to decrease with time in *Pc* infected mice and represents another measure of disease severity. For infected mice, the GEE analysis showed that SLE^s^ mice were consistently heavier than WT mice of the same pregnancy status at day 6, 7 and 8 post *Pc* infection. However, pregnant, infected SLE^s^ mice lost more weight between 6 and day 8 post *Pc* infection as compared to pregnant, infected WT mice (P<0.001). Again this effect appeared to be pregnancy related in that although the non-pregnant SLE^s^ mice were heavier than the WT mice, SLE^s^ and WT mice lost weight at the same rates between days 6 and 8 post *Pc* infection (P = 0.52) (Figure 1H).

Taken together these data indicate that as compared to pregnant WT mice, SLE^s^ pregnant mice had more severe symptoms of *Pc* infection, including higher parasitemias, lower hemoglobin levels and greater weight loss. Although malaria was more severe in pregnant SLE^s^ mice as compared to pregnant WT mice, SLE-susceptibility alone did not affect the severity of *Pc* malaria.

### Effects of SLE Susceptibility on Hematology Parameters

We also determined red blood cell counts, hematocrits, mean corpuscular volumes, platelet counts, white blood cell counts (WBC) and differential counts of leukocytes (basophils, eosinophils, neutrophils, lymphocytes, monocytes) before (day 0) and 8 days after *Pc* infection (day 8) (Figure S1). A linear regression with indicator variables for SLE status, malaria infection, and pregnancy status as covariates was performed to analyze the difference between day 0 and day 8 for each hematology parameter (Table S2). Controlling for infection and pregnancy status, SLE was significantly associated with the greater decrease in red blood cells, hematocrit, mean corpuscular volumes, and platelet counts between day 0 and day 8. The adjusted effect of malaria infection on the difference in eosinophils (P = 0.01), basophils (P<0.001), red blood cells (P<0.001) and hematocrit (P<0.001) was significant, while it was marginally significant on lymphocytes (P = 0.052), with infection being associated with a greater drop in all the above mentioned cell numbers. Finally, pregnancy is only significantly associated with an increase in mean corpuscular volume (P<0.001) after controlling for SLE-susceptibility and infection status.

### Effects of SLE Susceptibility on Reproduction

**Figure 2:**
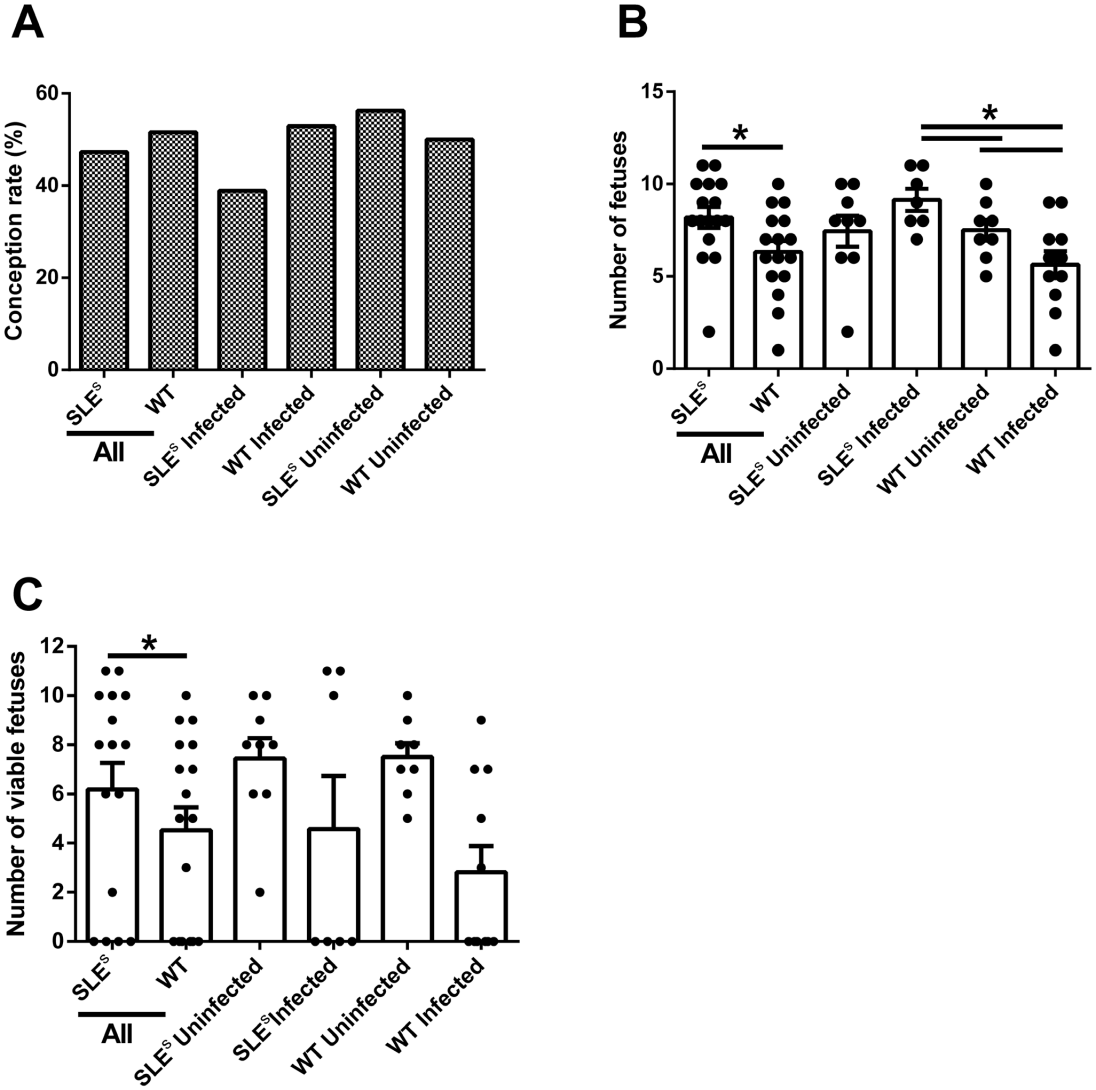
Effects of SLE susceptibility and malaria on reproductive fitness. (A) Conception rates (i.e. percentage of plugged animals that were determined to be pregnant) were determined at day 8 post-copula. (B) The total number of fetuses was determined at day 8 post-conception. (C) Graph shows the percentage of non-necrotic fetuses determined by analyzing H&E sections of uteri collected at day 8 post-conception. Samples marked as ‘All’ represented the pooled data for SLE^s^ or all WT, independent of infection status. WT (n = 36) and SLEs mice (N = 36).

We estimated the effects of SLE-susceptibility on pregnancy by measuring conception rates, defined as percent of inseminated animals that became pregnant. Differences in the conception rates of all SLE^s^ mice, infected or not, (44.4%) and of all WT mice (51.6%) was not statistically significant (P = 0.63 by Fisher’s exact test) (Figure 2A). A logistic regression analysis, adjusting for infection status, also failed to show significant effects of SLE^s^ on the conception rate (odds ratio [OR] = 0.77; 95% CI, 0.29–2.03; P = 0.60). However, the 95% confidence interval covers a fairly wide range of possibly decreased odds of conception rates. Given that the conception rate was approximately 50% in WT mice, we were underpowered to detect differences smaller than 35%.

We also analyzed the total number of fetuses (viable or not) and the number of viable fetus per pregnant SLE^s^ or WT mouse. Because mice will abort nonviable fetuses during the course of pregnancy, to allow an analysis of the effect of SLE-susceptibility on the total number of fetuses conceived we sacrificed all mice eight days after copula. The uteri were removed and the number of fetuses determined. A Poisson regression with robust standard error estimates was used to evaluate the data and showed that there was no significant difference in the total number of fetuses in uninfected SLE^s^ and WT mice (P = 0.96) (Figure 2B). Similarly, there was no significant difference in the number of viable fetuses in uninfected SLE^s^ and WT mice (P = 0.96) (Figure 2C). Thus, SLE susceptibility does not appear to have a reproductive cost.

### SLE Susceptibility is not Protective in PM

**Figure 3:**
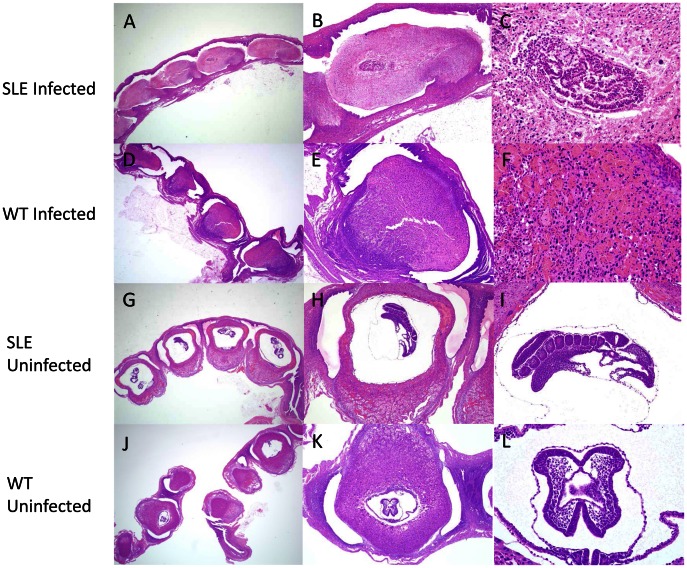
Malaria caused massive decidual necrosis in SLE^s^ and control animals. Uterine section of an infected SLE mouse (A) or infected WT mouse (D) showing massive decidual necrosis at day 8 post-conception at low-magnification (1.25×). High-magnification views (4× and 10×) of uteri from an infected SLE (B–C) and infected WT mouse (E–F). Uterine section of an uninfected SLE (G) or uninfected WT mouse (J) showing normal morphology of an 8 day old fetus at low-magnification (1.25×). High-magnification views (4× and 10×) of uteri from a non-infected SLE (H–I) or uninfected WT mouse (K–L). Uteri from all mice were analyzed by a pathologist (n = 72).

We evaluated the effect of SLE susceptibility on the number of viable fetuses in *Pc* infected mice. For both SLE^s^ and WT mice, *Pc* infection had a dramatic effect on the viability of fetuses (Figure 2C). The fetuses presented with massive necrosis but without obvious signs of exaggerated inflammation (Figure 3). Pathology due to *Pc* infection has not been reported this early in infection at a stage where the placenta is not completely formed and implantation is not complete. Infected SLE^s^ mice had 1.78 fold more fetuses per mouse as compared to infected WT mice (95% CI, 1.26–2.52; P = 0.001) (Figure 2B). In WT mice, *Pc* infection decreased the total number of fetuses by a factor of 1.46 (95% CI, 1.03–2.08; P = 0.04) (Figure 2B), while in SLEs mice the total number of fetuses increased by a factor of 1.23 (95% CI, 0.96–1.57, P = 0.10). *Pc* infection decreased the number of viable fetuses in SLE^s^ mice by a factor of 1.63 (95% CI, 0.66–4.05; P = 0.30) and in WT mice by a factor of 6.0 (95% CI, 1.52–23.7, P = 0.01) (Figure 2C). A Poisson regression with the logarithm of the total number of fetuses as the offset variable was fit to compare the proportion of viable fetuses between infected SLE^s^ and infected WT mice. The result using the robust standard errors suggested that SLE increased the proportion of viable fetuses by a factor of 2.05 (95% CI, 0.48–8.68; P = 0.33), although the difference was not statistically significant. The average number of viable fetuses in uninfected pregnant SLE mice was 7.4. With 9 uninfected pregnant SLE^s^ mice and 7 infected pregnant SLE^s^ mice, we had an 80% power to detect a reduction of 1.845 times fewer viable fetuses in infected pregnant SLE^s^ mice. Similarly, the average number of viable fetuses in uninfected pregnant WT mice is 7.5. With 8 uninfected pregnant WT mice and 8 infected pregnant WT mice, we had an 80% power to detect a reduction of 1.815 times fewer viable fetuses in infected pregnant WT mice.

## Discussion

The risk of SLE is significantly higher in African American women as compared to woman of European descent suggesting that the African genome contains SLE-susceptibility genes. To test the hypothesis that such genes were selected for in the African genome for their protective effect in severe malaria, we earlier determined the influence of genetic susceptibility to SLE on the outcome of CM in mice. We provided evidence that SLE-susceptibility did indeed protect mice from CM. Here we extended these studies to ask: does SLE-susceptibility have a cost in terms of reproductive fitness and does the protective effect of SLE-susceptibility to CM extend to another form of severe malaria, PM?

We show that independent of the infection status, SLE susceptibility did not affect conception, the total number of resulting fetus or fetal viability suggesting that SLE susceptibility does not have negative effects on fertilization, placentation, or the very early stages of pregnancy. Comparing the total number of fetuses by infection status we did not observe any differences, suggesting that malaria infection at the day of conception did not affect the formation of embryos.

We also observed that although *Pc* infections caused a significant reduction in the number of viable fetuses at day 8 post-conception in both SLE^s^ and WT mice. SLE^s^ mice, independent of their infection status, had more fetuses than WT but the number of viable fetuses was not different between infected SLE^s^ and WT mice. Overall, a larger number of fetuses with an equal number of viable ones equates to a positive effect on reproductive fitness. Interestingly, the effect was observed at day 8 post-conception, a point where the placentas were still forming and not completely implanted. The fetuses presented with massive necrosis without signs of exaggerated inflammation. To our knowledge, the effect of *Pc* infection on fetal health in pregnant mice has not been described at this early stage. Even though SLE^s^ mice were relatively protected from PM, infected, pregnant SLE^s^ mice had more serious malaria symptoms as compared to infected, pregnant WT mice. SLE^s^ mice lost more weight and had higher parasitemias and the lower hemoglobin levels suggesting that SLE may worsen the general response to malaria infection during pregnancy. SLE causes a variety of changes in the immune system that makes SLE patients more susceptible to infection. For instance, PMNs from SLE patients tend to be dysfunctional [14] and they are often neutropenic [15]. Furthermore, pregnancy by itself is immunosuppressive and capable of depressing PMN function [16]. As PMN activity correlates with clinical protection from malaria [17], it is possible that the worse response observed in SLE pregnant animals might be due to the combined detrimental effect of SLE and pregnancy on PMNs activity. Also, SLE patients tend to have lower levels of complement components, with C3 and C4 being considered markers of disease activity [18]. Complement activation and complement component levels also go down during malaria infection [19]. The decrease of certain complement components, as for instance C5 deficiency, is associated with higher levels of circulating inflammatory cytokines in response to infection [20], suggesting that SLE may aggravate systemic inflammation during infection. Interestingly, C5 deficient mice are protected against cerebral malaria [21]. Therefore, it is possible that decreased levels of some complement components in SLE individuals may increase susceptibility to pathogens while simultaneously protecting against pathology.

We also observed lower platelet counts in infected pregnant SLE^s^ versus WT mice at day 8. Thrombocytopenia is a common feature of SLE, pregnancy and malaria, with SLE thrombocytopenia thought to be caused by peripheral platelet destruction [22]. While gestational thrombocytopenia does not seem to affect maternal and fetal outcomes in pregnancy [23], in malaria patients thrombocytopenia has been associated with malaria severity and prognosis [24]. On the other hand, in SLE patients the effects are not clear, with conflicting reports suggesting no effect [22] or correlation with worse prognosis [25]. Recent data suggests that the pathogenesis of PM is mediated in part by an inflammation-coagulation cycle involving platelets as one of the key components in this cycle [26]. If so this suggests a potentially detrimental effect of SLE susceptibility on infected pregnant mice.

Taken together our results point toward a mixed effect of SLE susceptibility on PM. On the one hand, the malaria symptoms were worse in infected pregnant SLE^s^ mice as compared to WT mice but on the other hand, SLE^s^ mice produced more fetuses and a similar proportion of healthy fetuses during infection as compared to WT mice. However, overall the data presented here and our earlier results suggest that SLE susceptibility has a net positive effect on fitness as it can decrease mortality due to severe malaria and increase reproductive fitness by increasing the total number of fetuses.

The results presented here further support the hypothesis that genetic susceptibility to SLE is protective in severe malaria. However, we are still left with the paradox that genetic SLE susceptibility does not result in SLE in individuals living in malaria endemic areas of Africa. Two studies in mice suggest that malaria infections themselves may ‘recalibrate’ the inflammatory response in SLE-susceptible mice resulting in prevention of autoimmune disease [27], [28]. Future studies aimed at understanding the cellular and molecular interaction between the autoimmune susceptible host and the malaria parasite should inform us as to how inflammation can be controlled in both autoimmunity and in malaria.

## Supporting Information

Figure S1 **Effects of SLE and malaria on blood composition during pregnancy.** Animals were infected with 1×10^4^ *P. chabaudi* AS iRBCs on day 0 post-conception. Hematological parameters were determined in whole blood. White blood cells (WBC), platelets (PLT), lymphocytes (LY), eosinophils (EO), basophils (BA), hematocrit (HCT), red blood cells (RBC), mean corpuscular volume (MCV) and neutrophils (NE) and are shown. Data shows the median with the box upper and lower limits representing the upper and lower quartiles, the whiskers representing the range (maximum and minimum of the data) and open circles representing outliers.
(PDF)

Table S1 **P values for the adjusted effects of SLE and pregnancy on malaria infection at different time points using GEE models.** ¶ indicates analyses using uninfected mice.
(DOCX)

Table S2 **Adjusted effects (standard errors) of SLE, infection, and pregnancy on the difference between day 8 and day 0 in the hematology parameters using linear regressions.**
(DOCX)

## References

[1] Guyatt HL, Snow RW (2004) Impact of malaria during pregnancy on low birth weight in sub-Saharan Africa. Clin Microbiol Rev 17: 760–769, table of contents. 10.1128/CMR.17.4.760-769.2004 PMC523568 15489346

[2] Umbers AJ, Aitken EH, Rogerson SJ (2011) Malaria in pregnancy: small babies, big problem. Trends Parasitol 27: 168–175. 21377424 10.1016/j.pt.2011.01.007

[3] Brabin BJ, Romagosa C, Abdelgalil S, Menendez C, Verhoeff FH, et al (2004) The sick placenta-the role of malaria. Placenta 25: 359–378. 15081631 10.1016/j.placenta.2003.10.019

[4] Molokhia M, McKeigue P (2006) Systemic lupus erythematosus: genes versus environment in high risk populations. Lupus 15: 827–832. 17153858 10.1177/0961203306070007

[5] Bae SC, Fraser P, Liang MH (1998) The epidemiology of systemic lupus erythematosus in populations of African ancestry: a critical review of the “prevalence gradient hypothesis”. Arthritis Rheum 41: 2091–2099. 9870865 10.1002/1529-0131(199812)41:12<2091::AID-ART2>3.0.CO;2-D

[6] Greenwood B, Corrah T (2001) Systemic lupus erythematosus in African immigrants. Lancet 358: 1182. 10.1016/s0140-6736(01)06281-x 11597702

[7] Greenwood BM (1968) Autoimmune disease and parasitic infections in Nigerians. Lancet 2: 380–382. 4174413 10.1016/s0140-6736(68)90595-3

[8] Butcher GA, Clark IA (1990) SLE and malaria: another look at an old idea. Parasitol Today 6: 259–261. 15463357 10.1016/0169-4758(90)90186-8

[9] Bolland S, Yim YS, Tus K, Wakeland EK, Ravetch JV (2002) Genetic modifiers of systemic lupus erythematosus in FcgammaRIIB(−/−) mice. J Exp Med 195: 1167–1174. 11994421 10.1084/jem.20020165 PMC2193704

[10] Rest JR (1982) Cerebral malaria in inbred mice. I. A new model and its pathology. Trans R Soc Trop Med Hyg 76: 410–415. 7051459 10.1016/0035-9203(82)90203-6

[11] Waisberg M, Tarasenko T, Vickers BK, Scott BL, Willcocks LC, et al (2011) Genetic susceptibility to systemic lupus erythematosus protects against cerebral malaria in mice. Proc Natl Acad Sci U S A 108: 1122–1127. 21187399 10.1073/pnas.1017996108 PMC3024697

[12] Willcocks LC, Carr EJ, Niederer HA, Rayner TF, Williams TN, et al (2010) A defunctioning polymorphism in FCGR2B is associated with protection against malaria but susceptibility to systemic lupus erythematosus. Proc Natl Acad Sci U S A 107: 7881–7885. 20385827 10.1073/pnas.0915133107 PMC2867866

[13] Poovassery J, Moore JM (2006) Murine malaria infection induces fetal loss associated with accumulation of Plasmodium chabaudi AS-infected erythrocytes in the placenta. Infect Immun 74: 2839–2848. 16622222 10.1128/IAI.74.5.2839-2848.2006 PMC1459757

[14] Wu SA, Yeh KW, Lee WI, Yao TC, Kuo ML, et al (2013) Impaired phagocytosis and susceptibility to infection in pediatric-onset systemic lupus erythematosus. Lupus 22: 279–288. 23369850 10.1177/0961203312474704

[15] Lee SW, Park MC, Lee SK, Park YB (2012) Adjusted neutropenia is associated with early serious infection in systemic lupus erythematosus. Mod Rheumatol. 10.1007/s10165-012-0666-1 22678568

[16] Crocker I, Lawson N, Daniels I, Baker P, Fletcher J (1999) Significance of fatty acids in pregnancy-induced immunosuppression. Clin Diagn Lab Immunol 6: 587–593. 10391868 10.1128/cdli.6.4.587-593.1999 PMC95733

[17] Joos C, Marrama L, Polson HE, Corre S, Diatta AM, et al (2010) Clinical protection from falciparum malaria correlates with neutrophil respiratory bursts induced by merozoites opsonized with human serum antibodies. PLoS One 5: e9871. 20360847 10.1371/journal.pone.0009871 PMC2845614

[18] Sturfelt G, Truedsson L (2005) Complement and its breakdown products in SLE. Rheumatology (Oxford) 44: 1227–1232. 15972354 10.1093/rheumatology/keh719

[19] Phanuphak P, Hanvanich M, Sakulramrung R, Moollaor P, Sitprija V, et al (1985) Complement changes in falciparum malaria infection. Clin Exp Immunol 59: 571–576. 3886219 PMC1576927

[20] Mullick A, Elias M, Picard S, Bourget L, Jovcevski O, et al (2004) Dysregulated inflammatory response to Candida albicans in a C5-deficient mouse strain. Infect Immun 72: 5868–5876. 15385488 10.1128/IAI.72.10.5868-5876.2004 PMC517586

[21] Patel SN, Berghout J, Lovegrove FE, Ayi K, Conroy A, et al (2008) C5 deficiency and C5a or C5aR blockade protects against cerebral malaria. J Exp Med 205: 1133–1143. 18426986 10.1084/jem.20072248 PMC2373845

[22] Ziakas PD, Giannouli S, Zintzaras E, Tzioufas AG, Voulgarelis M (2005) Lupus thrombocytopenia: clinical implications and prognostic significance. Ann Rheum Dis 64: 1366–1369. 16100344 10.1136/ard.2004.033100 PMC1755663

[23] Burrows RF, Kelton JG (1993) Fetal thrombocytopenia and its relation to maternal thrombocytopenia. N Engl J Med 329: 1463–1466. 8413457 10.1056/NEJM199311113292005

[24] Gerardin P, Rogier C, Ka AS, Jouvencel P, Brousse V, et al (2002) Prognostic value of thrombocytopenia in African children with falciparum malaria. Am J Trop Med Hyg 66: 686–691. 12224575 10.4269/ajtmh.2002.66.686

[25] Jallouli M, Frigui M, Marzouk S, Snoussi M, Kechaou M, et al (2012) Clinical implications and prognostic significance of thrombocytopenia in Tunisian patients with systemic lupus erythematosus. Lupus 21: 682–687. 22354537 10.1177/0961203312438630

[26] Francischetti IM, Seydel KB, Monteiro RQ (2008) Blood coagulation, inflammation, and malaria. Microcirculation 15: 81–107. 18260002 10.1080/10739680701451516 PMC2892216

[27] Hentati B, Sato MN, Payelle-Brogard B, Avrameas S, Ternynck T (1994) Beneficial effect of polyclonal immunoglobulins from malaria-infected BALB/c mice on the lupus-like syndrome of (NZB x NZW)F1 mice. Eur J Immunol 24: 8–15. 8020574 10.1002/eji.1830240103

[28] Greenwood BM, Voller A (1970) Suppression of autoimmune disease in NZB and NZB-NZW F1 hybrid mice by infection with malaria. Trans R Soc Trop Med Hyg 64: 7. 5309735

