# Supplementary figures and images for: The Impact of Genetic Susceptibility to Systemic Lupus Erythematosus on Placental Malaria in Mice
Source: PLoS One. 2013 May 10;8(5):e62820. doi: 10.1371/journal.pone.0062820 (PMC3651086; doi:10.1371/journal.pone.0062820)

Figure S1

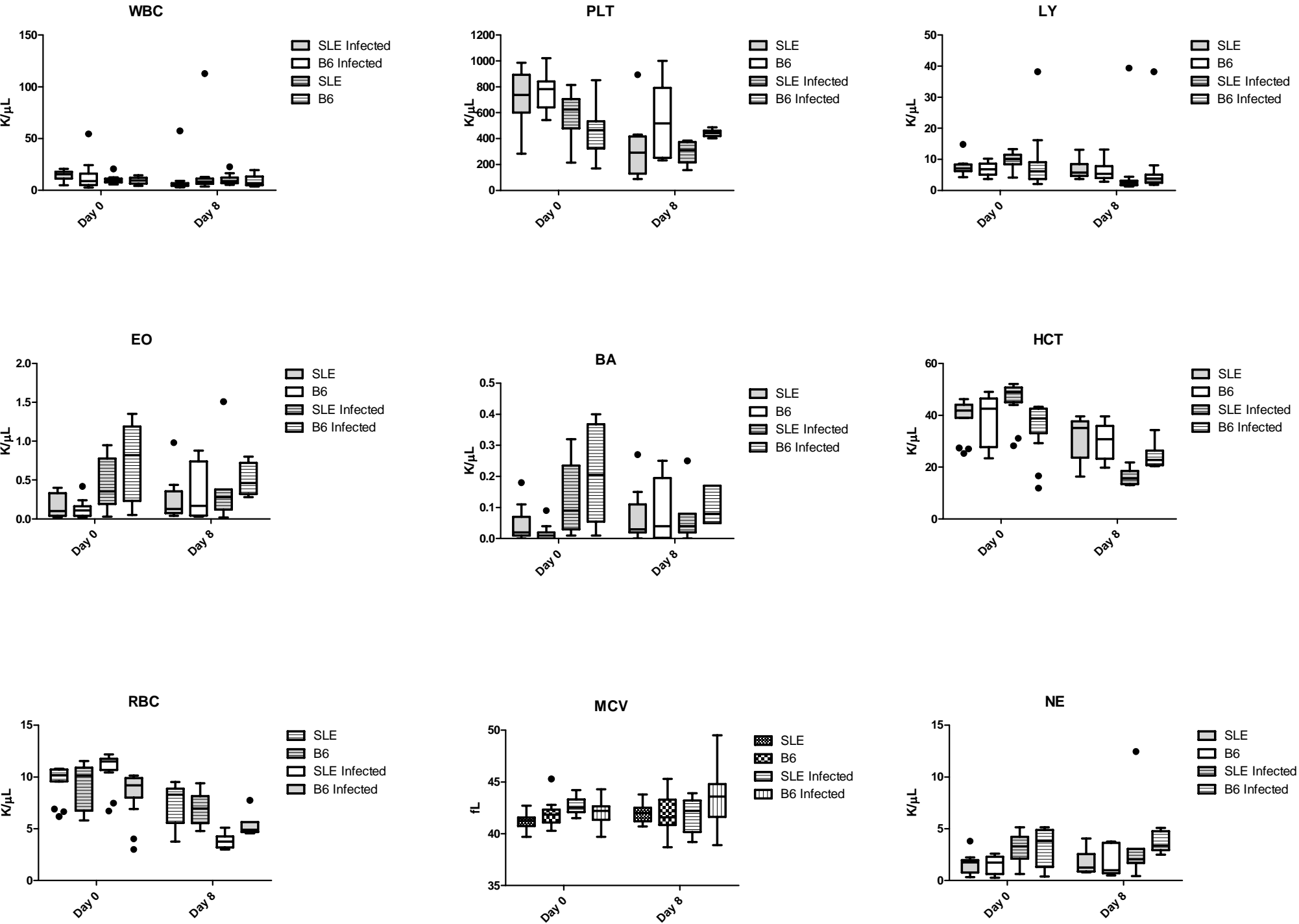

Supplement: Figure S1 — Effects of SLE and malaria on blood composition during pregnancy. Animals were infected with 1×10^4^ P. chabaudi AS iRBCs on day 0 post-conception. Hematological parameters were determined in whole blood. White blood cells (WBC), platelets (PLT), lymphocytes (LY), eosinophils (EO), basophils (BA), hematocrit (HCT), red blood cells (RBC), mean corpuscular volume (MCV) and neutrophils (NE) and are shown. Data shows the median with the box upper and lower limits representing the upper and lower quartiles, the whiskers representing the range (maximum and minimum of the data) and open circles representing outliers. (PDF) [file pone.0062820.s001.pdf]
