# Supplementary material for: The Impact of Genetic Susceptibility to Systemic Lupus Erythematosus on Placental Malaria in Mice
Source: PLoS One. 2013 May 10;8(5):e62820. doi: 10.1371/journal.pone.0062820 (PMC3651086; doi:10.1371/journal.pone.0062820)
Supplement: Table S1 — P values for the adjusted effects of SLE and pregnancy on malaria infection at different time points using GEE models. ¶ indicates analyses using uninfected mice. (DOCX) [file pone.0062820.s002.docx]

Table S1 – *P* values for the adjusted effects of SLE and pregnancy on malaria infection at different time points using GEE models. ¶ indicates analyses using uninfected mice.

|  | Comparisons at Day 6 | | | |
| --- | --- | --- | --- | --- |
|  | Pregnant | Non-pregnant | SLE | B6 |
|  | SLE vs B6 | SLE vs B6 | Pregnant vs non-pregnant | Pregnant vs non-pregnant |
| Parasitemia | 0.004 | 0.34 | 0.06 | 0.81 |
| Hemoglobin | 0.01 | <0.001 | 0.96 | 0.45 |
| Hemoglobin^¶^ | 0.28 | 0.44 | 0.92 | 0.87 |
| Weight | <0.001 | 0.02 | 0.01 | 0.13 |
| Weight^¶^ | 0.13 | 0.004 | 0.33 | 0.01 |

|  | Comparisons at Day 7 | | | |
| --- | --- | --- | --- | --- |
|  | Pregnant | Non-pregnant | SLE | B6 |
|  | SLE vs B6 | SLE vs B6 | Pregnant vs unpregnant | Pregnant vs unpregnant |
| Parasitemia | 0.01 | 0.06 | 0.16 | 0.65 |
| Hemoglobin | 0.003 | 0.12 | 0.08 | 0.89 |
| Hemoglobin^¶^ | 0.12 | 0.98 | 0.02 | 0.45 |
| weight | <0.001 | 0.02 | 0.02 | 0.76 |
| Weight^¶^ | 0.10 | 0.003 | 0.21 | 0.01 |

|  | Comparisons at Day 8 | | | |
| --- | --- | --- | --- | --- |
|  | Pregnant | Non-pregnant | SLE | B6 |
|  | SLE vs B6 | SLE vs B6 | Pregnant vs non-pregnant | Pregnant vs nonpegnant |
| Parasitemia | 0.04 | 0.07 | 0.13 | 0.28 |
| Hemoglobin | <0.001 | 0.02 | 0.01 | 0.33 |
| Hemoglobin^¶^ | 0.53 | 0.97 | 0.04 | 0.01 |
| Weight | 0.003 | 0.06 | 0.74 | 0.37 |
| Weight^¶^ | 0.11 | 0.002 | 0.13 | 0.001 |
