# Supplementary material for: The Impact of Genetic Susceptibility to Systemic Lupus Erythematosus on Placental Malaria in Mice
Source: PLoS One. 2013 May 10;8(5):e62820. doi: 10.1371/journal.pone.0062820 (PMC3651086; doi:10.1371/journal.pone.0062820)
Supplement: Table S2 — Adjusted effects (standard errors) of SLE, infection, and pregnancy on the difference between day 8 and day 0 in the hematology parameters using linear regressions. (DOCX) [file pone.0062820.s003.docx]

Table S2 – Adjusted effects (standard errors) of SLE, infection, and pregnancy on the difference between day 8 and day 0 in the hematology parameters using linear regressions.

| Change in hematology parameters | Intercept | | SLE | Infection | Pregnancy |
| --- | --- | --- | --- | --- | --- |
| WBC | -0.36  (2.41) | | -1.40  (2.43) | -4.00  (2.44) | 1.60  (2.42) |
| NEU | 0.08  (0.61) | | -0.11  (0.61) | -0.48  (0.61) | 0.42  (0.61) |
| LYM | -0.62  (1.58) | | -1.24  (1.59) | -3.12  (1.59) | 0.93  (1.58) |
| MON | 0.02  (0.20) | | -0.15  (0.21) | 0.05  (0.21) | 0.28  (0.20) |
| EOS | 0.12  (0.11) | | 0.08  (0.11) | -0.31*  (0.11) | 0.01  (0.11) |
| BAS | 0.05  (0.03) | | 0.03  (0.03) | -0.14*  (0.03) | -0.02  (0.03) |
| PLT | -189*  (65) | | -204*  (63) | 84  (63) | 3  (63) |
| RBC | -0.42  (0.75) | | -1.48*  (0.72) | -2.79*  (0.73) | -1.26  (0.73) |
| HTC | -1.59  (3.27) | -6.99*  (3.18) | | -11.91*  (3.18) | -4.26  (3.18) |
| MCV | 0.25  (0.47) | -1.34*  (0.46) | | 0.34  (0.46) | 1.71*  (0.46) |
